# Reviving the Skin From Within: Mechanistic Insights Into a Well‐Tolerated Dermal Filler—CPM‐HA20G

**DOI:** 10.1111/jocd.70996

**Published:** 2026-06-18

**Authors:** Kay Marquardt, Christian Hartmann, Sonja Sattler, Je‐Young Park, Ting Song Lim, Luis Alberto Parra Hernandez, Christina Wollenburg, Daniela Schäfer, Sarah Hetier‐Jaccoud, Thomas Hengl

**Affiliations:** ^1^ R&D Skin Lab & Nonclinical Science Department Merz Aesthetics GmbH Frankfurt am Main Germany; ^2^ Rosenpark Klinik Darmstadt Germany; ^3^ Apkoo‐Jung Oracle Dermatology Center Seoul Republic of Korea; ^4^ Clique Clinic Kuala Lumpur Malaysia; ^5^ Sociedad Internacional de Rejuvenecimiento Facial no quirúrgico (SIRF) Barranquilla Colombia; ^6^ Merz Aesthetics Raleigh North Carolina USA

**Keywords:** CPM‐HA20G, dermal filler, glycerol, hyaluronic acid, hydration, inflammation, skin

## Abstract

**Background:**

Dermal fillers composed of hyaluronic acid (HA) combined with glycerol have been clinically shown to enhance skin hydration and quality; the precise mechanisms underlying their efficacy have yet to be fully elucidated.

**Aims:**

Investigation of the dual hydration mechanisms, cell protection and tolerability, and tissue compatibility of the cohesive polydensified matrix (CPM) filler containing HA and glycerol (CPM‐HA20G), focusing on glycerol's unique role.

**Methods:**

Primary normal human epidermal keratinocytes, in vitro skin, ex vivo human skin models, and an in vivo study were used to evaluate CPM‐HA20G, with additional assessments of its humectant, glycerol. Comparisons were further made with a cross‐linked HA filler containing sorbitol (HA12.5S) indented for similar use, as well as with the humectants sorbitol and mannitol. Gene expression analysis, protein analysis, cell‐based assays, 3D micro‐computed tomography (μCT), TEWL, corneometry, and histology were performed to assess aquaporin‐3 (AQP3) modulation, cellular stress levels, protection from UVB—or H_2_O_2_‐induced oxidative stress, hydration dynamics, tissue integration, and inflammation.

**Results:**

CPM‐HA20G immediately increased hydrated tissue volumes by 40% within 10 min and 63% after 120 min post‐injection, as measured via μCT indicating improved inner tissue hydration. Progressively, CPM‐HA20G increased surface hydration values from the inside out, whereas the glycerol component of CPM‐HA20G contributing a pronounced hydration effect. Glycerol uptake in keratinocytes occurred without upregulating AQP3 expression, in contrast to sorbitol and mannitol, which triggered AQP3 overexpression (*p* < 0.001), along with the cellular stress marker, phosphorylation of P38 (pP38) (*p* < 0.0001) and reduced keratinocyte viability (*p* < 0.001, *p* < 0.01). Similarly, AQP inhibition by DFP00173 or phloretin elicited a comparable P38 stress response, which was attenuated by glycerol treatment. Compared to sorbitol and mannitol, glycerol demonstrated well cellular tolerability. Glycerol treatment reduced UVB—or H_2_O_2_‐induced reactive oxygen species (ROS) in keratinocytes by 68% (*p* < 0.0001), highlighting its antioxidative capacity. Furthermore, histological analysis verified improved tissue integration and reduced inflammatory response for CPM‐HA20G relative to the HA‐filler HA12.5S containing sorbitol.

**Conclusions:**

This preclinical study demonstrates that CPM‐HA20G achieves rapid tissue hydration via a dual hydration mechanism and is well tolerable with favorable tissue integration. Glycerol was found to play a unique role in enhancing hydration without inducing stress responses, providing antioxidative capacity and cell protection. These findings provide a mechanistic basis for the clinical efficacy of CPM‐HA20G.

**Abbreviations**
AQP: aquaporin
CPM: cohesive polydensified matrix
HA: hyaluronic acid
HU: Hounsfield units
IHC: immunohistochemistry
NHEKs: normal human epidermal keratinocytes
NMF: natural moisturizing factor
ROS: reactive oxygen species
SC: stratum corneum
SHE: safranin‐hematoxylin‐eosin
TEWL: Transepidermal water loss
μCT: micro‐computed tomography

## 1 Introduction

Skin quality is a foundational element of facial attractiveness and a primary motivator for seeking aesthetic treatments. A recent international consensus defines good skin quality as healthy, undamaged, and youthful in appearance; attributes recognized across all ethnicities and age groups [1]. The condition of the skin significantly influences perceptions of age, health, and attractiveness, with even minor changes in surface texture or pigmentation having a pronounced impact on how a face is perceived [1].

The Cohesive Polydensified Matrix hyaluronic acid dermal filler (CPM‐HA20G, Belotero Revive, Anteis S.A., Plan‐les‐Ouates, Switzerland, a company of the Merz Aesthetics group) is formulated with glycerol and the CPM technology. While hyaluronic acid (HA) has long been used in fillers for its viscoelastic and hydrating properties, glycerol introduces a biological component with distinct and complementary mechanisms of action relevant to skin physiology. Its dual‐action formula rehydrates dry to very dry skin and smooths superficial fine lines, distinguishing it from conventional HA‐based products [2].

Glycerol, a naturally occurring polyol, is endogenously present in human skin, where it contributes to stratum corneum (SC) hydration, barrier integrity, and lipid organization. Glycerol uptake into epidermal cells occurs primarily through glycerol permeable Aquaporin‐3 (AQP3) channels, an aquaglyceroporin abundantly expressed in the basal and spinous layers of the epidermis [3]. AQP3 facilitates the transport of glycerol and water, playing a key role in skin hydration, elasticity, and barrier recovery. Functionally, AQP3‐mediated glycerol transport supports keratinocyte proliferation, migration, and wound healing—highlighting its multifaceted contribution to epidermal homeostasis [4]. Through its three hydroxyl groups, glycerol readily forms hydrogen bonds with water, acting as a humectant by attracting and retaining water molecules from both the surrounding environment and underlying dermal layers [5]. Additionally, glycerol supports the SC lipid matrix by stabilizing its liquid crystalline phase and preventing phase transitions that lead to barrier dysfunction [6]. Glycerol is well known for its benefits as a topical agent; however, intradermal injection in combination with a dermal HA filler requires further investigation.

Clinical studies with CPM‐HA20G have demonstrated significant and sustained improvements in skin hydration, elasticity, firmness, and surface texture in patients with early signs of face aging [7]. Objective biophysical measurements confirmed that these clinical effects persisted for up to 36 weeks post‐treatment, with high levels of subject and investigator satisfaction, and an excellent safety profile.

The present investigation aims to characterize the molecular and cellular effects of glycerol when delivered through the CPM‐HA20G formulation, with a particular focus on its capacity to enhance skin quality through dual hydration mechanisms:

Inner Hydration: Characterized by immediate water‐binding effects within the dermal matrix and Inside‐Out Hydration: Mediated by glycerol transport into the epidermis, resulting in long‐term improvements in hydration.

Comparisons were made to investigate the cell tolerability and anti‐inflammatory activity of glycerol versus other commonly used humectants. Finally, the performance of CPM‐HA20G was assessed in comparison to a dermal filler using the humectant sorbitol in terms of inflammatory reaction and tissue integration.

## 2 Materials and Methods

### 2.1 Cell Culture

Normal Human Epidermal Keratinocytes (NHEKs) (Promocell, C‐12003, Germany) were cultured at 37°C in T75 Nunc flasks with keratinocyte growth medium (Promocell, C‐20011, Germany) supplemented with 100 units/mL penicillin and 100 μg/mL streptomycin (Cytiva, SV30010, United States).

### 2.2 Cell Count and Viability

NHEKs were treated with glycerol (Sigma, #SHBL0454, Germany), sorbitol (Sigma, #3037C002, Germany), or mannitol (Merck, #M4125) (diluted in normal growth medium) at 95 mM–190 mM for either 24, 48, or 72 h. The osmolarity of substances was measured using OM807 (Vogel, Germany) to ensure that osmolarity was in the same range (Table S1). Cellular viability was evaluated using a Resazurin cell viability assay (Abcam, ab129732, England), following the manufacturer's protocol. Cell count was analyzed by using a nuclear stain (DAPI).

### 2.3 Glycerol Uptake Assay

The Glycerol‐Glo (Promega, J3151, Germany) assay was performed following the manufacturer's instructions. NHEKs were incubated with 95 or 190 mM of glycerol for 2, 24, 48, or 72 h. Luminescence was recorded using a plate‐reading luminometer.

### 2.4 Inhibition of AQP Channels and Associated Stress Response

NHEKs were pretreated with DFP00173 50–100 μM (DFP, TargetMol, TGM‐T11014, United States), a known AQP3 inhibitor [8], or Phloretin 250 μM (Sigma, P7912, Germany), a general AQP inhibitor [9, 10, 11] for 1 h. After pre‐incubation with inhibitors, medium was exchanged for co‐treatment with media containing the respective inhibitors and glycerol at either 95 mM or 190 mM with subsequent incubation for 2 h. Samples were then lysed in NP40 lysis buffer and analyzed by western blotting for associated stress responses via pP38/tP38. As both inhibitors are solved in DMSO, all samples were incubated with similar DMSO concentration (0.5%) throughout the experiment, and 0.5% DMSO treatment was used as control condition.

### 2.5 Gene Expression Analysis by qPCR

RNA was isolated using a Qiagen RNA isolation kit (Qiagen, 73 404, Germany). RNA yield was subsequently quantified by Agilent RNA 6000 Nano Kit (Agilent, 5067‐1511, United States) following the manufacturer's instructions. cDNA was synthesized from 1 μg of the isolated RNA using the Reverse Transcription Kit (Qiagen, 205 311, Germany). All real‐time PCR reactions were performed in triplicate on an Applied Biosystems 7500 Real‐Time PCR System using predesigned TaqMan Real‐Time PCR assays (Thermo Fisher Scientific, Germany). mRNA levels were normalized to the expression of housekeeping genes, and relative expression levels were calculated as ∆∆Ct. TaqMan PCR primers (Thermo Fisher Scientific, Germany): B2M (Hs99999907_m1), RPL13A (Hs083043885_g1), and *AQP3 (Hs00185020)* were used.

### 2.6 Protein Expression Analysis by Western Blot

NHEKs were lysed using NP‐40 cell lysis buffer with added protease inhibitor (1×) and 0.1% sodium dodecyl sulfate. The lysates were prepared using a cell scraper, incubated for 30 min at 4°C, and then centrifuged at 13 000 × *g* for 30 min. The supernatants were stored in Eppendorf tubes at −20°C.

Standard reagents and sample preparation were completed according to the Biotechne Abby protocol. Samples were incubated for 5 min at 95°C and then placed on ice to cool. Samples and reagents were loaded into a microplate, and the proteins were separated by Protein Simple (Abby, Biotechne, United States). For the detection of protein abundance, the following antibodies were used: phosphorylated P38 (Cell Signaling, 8690, United States) and total P38 (Cell Signaling, 4511S, United States). Beta‐tubulin (Merck, T8328) or GAPDH (Thermo Fisher, PA1‐987) was used as a loading control. Detection of the proteins was performed using a chemiluminescent reaction with secondary antibodies anti‐mouse or anti‐rabbit (Biotechne, DM002, DM001). The luminescence signal was analyzed by the software “Compass for Simple Western,” and the results with target proteins were normalized to the total amount of proteins loaded.

### 2.7 Immunocytochemistry

NHEKs were seeded in ibidi μ‐Slide 8‐well chambers (Ibidi, 80 826, Germany). After Treatment, the cells were fixed with 4% paraformaldehyde (PFA), followed by wash steps with phosphate‐buffered solution (PBS) and permeabilization with 0.5% Triton X‐100. Samples were treated with blocking solution (ROTI Immunoblock) (Carl Roth, T144.1, Germany) for 2 h at room temperature (RT). Samples were incubated overnight at 4°C with primary antibodies against phosphorylated‐P38, total P38, and AQP3. Secondary antibodies conjugated to fluorophores were used to visualize the target proteins: anti‐rabbit antibodies, Alexa Fluor 488 goat anti‐rabbit (2 mg/mL; Thermo Fisher Scientific, #A‐11008) with a 488 nm fluorophore and anti‐mouse antibodies with a 568 nm fluorophore, Alexa Fluor 568 goat anti‐mouse (2 mg/mL; Thermo Fisher Scientific, #A‐11004). DAPI (2 μg/mL, Thermo Fisher, #D21490) and Cellmask staining were added to visualize the cell cytoplasm. The samples were analyzed using an inverted Axio Observer 7 (Carl Zeiss, Germany) with Zen Blue software.

### 2.8 ROS Assay

NHEKs were seeded in 96‐well plates and cultured to confluence. Dichlorodihydrofluorescein diacetate (DCF‐DA, Abcam, #ab113851) was applied to the cells and then exposed to either UVB (100 mJ/cm^2^) or hydrogen peroxide (H_2_O_2_) (200 μM). Fluorescence was measured (excitation, 485 nm; emission, 518 nm) using a microplate reader. Cells were pretreated with glycerol (190 mM–9.5 mM) for 24 h prior to stressor exposure (pretreatment) or treated immediately after stressor exposure (post‐treatment). Negative control groups were untreated, while positive control groups were treated with Trolox (Merck, #648471, Germany). Baseline reactive oxygen species (ROS) generation was determined in cells not exposed to UVB or H_2_O_2_. Mean fluorescence intensity (relative fluorescence units, RFUs) was calculated for each group and compared by ANOVA.

### 2.9 Tissue Hydration Analysis by Micro CT (μCT)

HypoSkin human skin model (Genoskin, Toulouse, France) of Caucasian skin (Fitzpatrick III) was used in this study, with informed consent from donors and in compliance with the Declaration of Helsinki and all relevant institutional ethical guidelines. Subcutaneous bolus injection of CPM‐HA20G was delivered via 30G × ½″ needle at 44 ± 19 μL/cm^2^ without post‐injection massage. Hydration dynamics were quantified using non‐invasive micro‐computed tomography (μCT; SkyScan 1278, Bruker) with the following parameters: resolution, 20 μm/voxel; voltage, 65 kV; filter, 0.25 mm aluminum; and rotation step, 0.4°. Skin models were maintained in proprietary culture medium at 37°C with ~95% relative humidity throughout the 0–2 h observation period.

### 2.10 Skin Surface Hydration Analysis by Corneometer

HypoSkin human skin model (Genoskin, Toulouse, France) of Caucasian skin (Fitzpatrick III) was used to compare the surface hydration effects after a 100 μL intradermal bolus injection of 5.4 M sorbitol, 5.4 M glycerol, PBS, or CPM‐HA20G over a time period of maximal 5 days, keeping the models hydrated from the basal orientation by growth medium. Hydration was measured using a Corneometer CM 825 (Courage & Khazaka, Cologne, Germany).

For dehydrated skin model experiments, HypoSkin human skin model (Genoskin, Toulouse, France) of Caucasian skin (Fitzpatrick III) was incubated at 40% rel. humidity with no growth medium. 150 μL of either CPM‐HA20G, 40% wt/wt glycerol, or PBS was intradermally injected into the skin. Surface hydration was measured using a Corneometer CM 825.

### 2.11 Transepidermal Water Loss Analysis (TEWL)

Five days old reconstructed human epidermis (RHE) models were systemically treated with CPM‐HA20G at 1% or 10% or glycerol at 9.5 mM or 95 mM. In addition, RHE models were cultured either under control standard condition (~95% rel. humidity) or low (~25%) humidity for 5 consecutive days (D5‐D10) to reflect a dry skin condition. Treatment of CPM‐HA20G and glycerol was renewed each day (D5‐D10). TEWL of RHE models was measured using a Tewameter TM300 (Courage & Khazaka, Germany) according to the manufacturer's instructions. TEWL was measured in biological triplicates; for each reading replicate, 30 measurements were collected (1 measurement/s), and the mean of these measurements expressed in g/m^2^/h was used for quantification.

### 2.12 In Vivo Analysis of Tissue Integration and Inflammation

Tissue integration and inflammation were investigated in an in vivo study (New Zealand White Rabbit). The study was conducted in an accredited AAALAC facility with authorization from the ethical committee (APAFIS #23401–2 017 050 517 312 149 v12). Intradermal bolus injections (200 ± 40 μL) were done with CPM‐HA20G (*n* = 12) and HA12.5S (*n* = 13), a commercially available dermal filler containing 12.5 mg/g cross‐linked and free hyaluronic acid and sorbitol. Post treatment, tissue was fixed in 10% neutral buffered formalin (NBF); sections were dehydrated with alcohol, cleared in xylene, and embedded in paraffin. Safranin Hematoxylin and eosin (SHE) stained central cross‐section (~4.5 μm) at the injection bolus was evaluated in terms of tissue integration and inflammation by a DESV‐Pathology using a scoring system of 0 (none) to 4 (complete tissue integration) or in terms of reactivity/inflammation score: 0.0–2.9 (no reaction), 3.0–8.9 (slight reaction), 9.0–15.0 (moderate reaction), and ≥ 15.1 (severe reaction). In addition, representative sections were analyzed for number of fiber segments in the bolus area using ImageJ [12].

### 2.13 Statistical Analysis

Statistical analysis was performed using one‐way ANOVA and subsequent post hoc test, with single pooled variance. *p*‐values < 0.05 were considered statistically significant. All experiments were conducted at least in triplicates or otherwise stated. Statistical analyses were conducted in GraphPad Prism (GraphPad Software, San Diego, USA).

## 3 Results

### 3.1 CPM‐HA20G Increases Hydration in the Skin

To investigate hydration dynamics following dermal filler administration, non‐invasive μCT was used to quantify radiation absorption and attenuation coefficients in human skin specimens. Baseline μCT signals (Hounsfield units, HU) of the analyzed materials prior to injection are summarized in Table 1.

**TABLE 1:** Hounsfield units (HU) of different samples measured by μCT acquisition.

Sample	Mean density (HU)	SD (HU)
Epidermis/Dermis	7.9	26.2
Hypodermis	−313.6	24.6
Cell Culture medium	71.6	22.2
CPM‐HA20G	20.2	21.1

**FIGURE 1:**
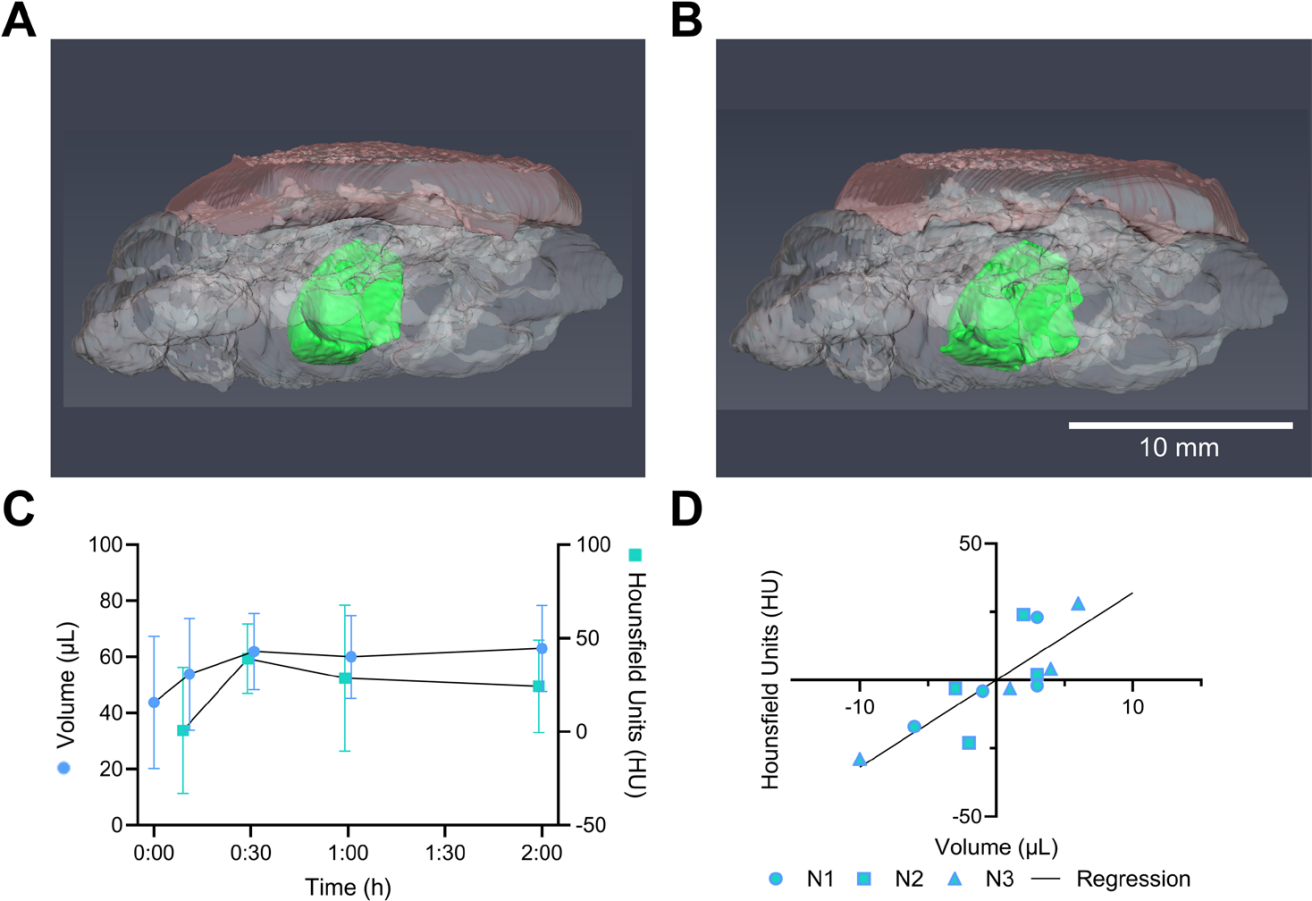
Tissue hydration at the injection site after filler injection. (A) 3D computer tomography analysis after subcutaneous bolus injection of CPM‐HA20G at 10 min and (B) at 120 min post‐injection in a human skin model. Injection site: Green; Hypodermis: Gray; Epidermis/Dermis: Red. Scale: 10 mm. Experiments were performed in biological triplicates (*n* = 3). (C) Correlation between volume and Hounsfield units (HU) at the injection site. (D) Within‐subject relationship between volume and HU after removal of replicate‐specific offsets, including the regression line with a slope of 3.12 HU/μL.

Analysis of subcutaneous injection of CPM‐HA20G into hydrated tissue showed distinct alterations in the HU values at the injection site compared to adjacent regions (Figure 1A,B, green, Video S1). Over time, HU values in the hypodermis remained within the expected physiological range (−110 to −350 HU), whereas marked changes were observed at the injection sites, with HU values ranging from −37 to 67 HU. The first post‐injection measurement at 10 min showed a mean HU of 1 ± 33, followed by a peak of 39 ± 19 HU at 30 min and stabilization at 25 ± 25 HU by 120 min (Figure 1C). Volumetric analysis revealed a time‐dependent increase similar to the HU density profile, with an increase of 32% ± 20% at 10 min, peaking at 30 min and reaching a plateau of 63% ± 53% by 120 min, demonstrating rapid hydration within the first post‐injection hour. A regression analysis was performed to determine whether there was a correlation between volume expansion correlated with HU changes. As the three injections exhibited markedly different baseline offsets of −37, 11, and 28 HU, a dummy‐adjusted multiple linear regression model was used (Figure 1D). This revealed a significantly positive association with a slope of 3.12 HU per volume (SE = 0.837, *p* = 0.0059) and explained 87% of the variance (R^2^ = 0.865). These results demonstrate that HU values increase significantly with increasing volume.

### 3.2 CPM‐HA20G Increases Surface Hydration and Transepidermal Water Loss of Skin

**FIGURE 2:**
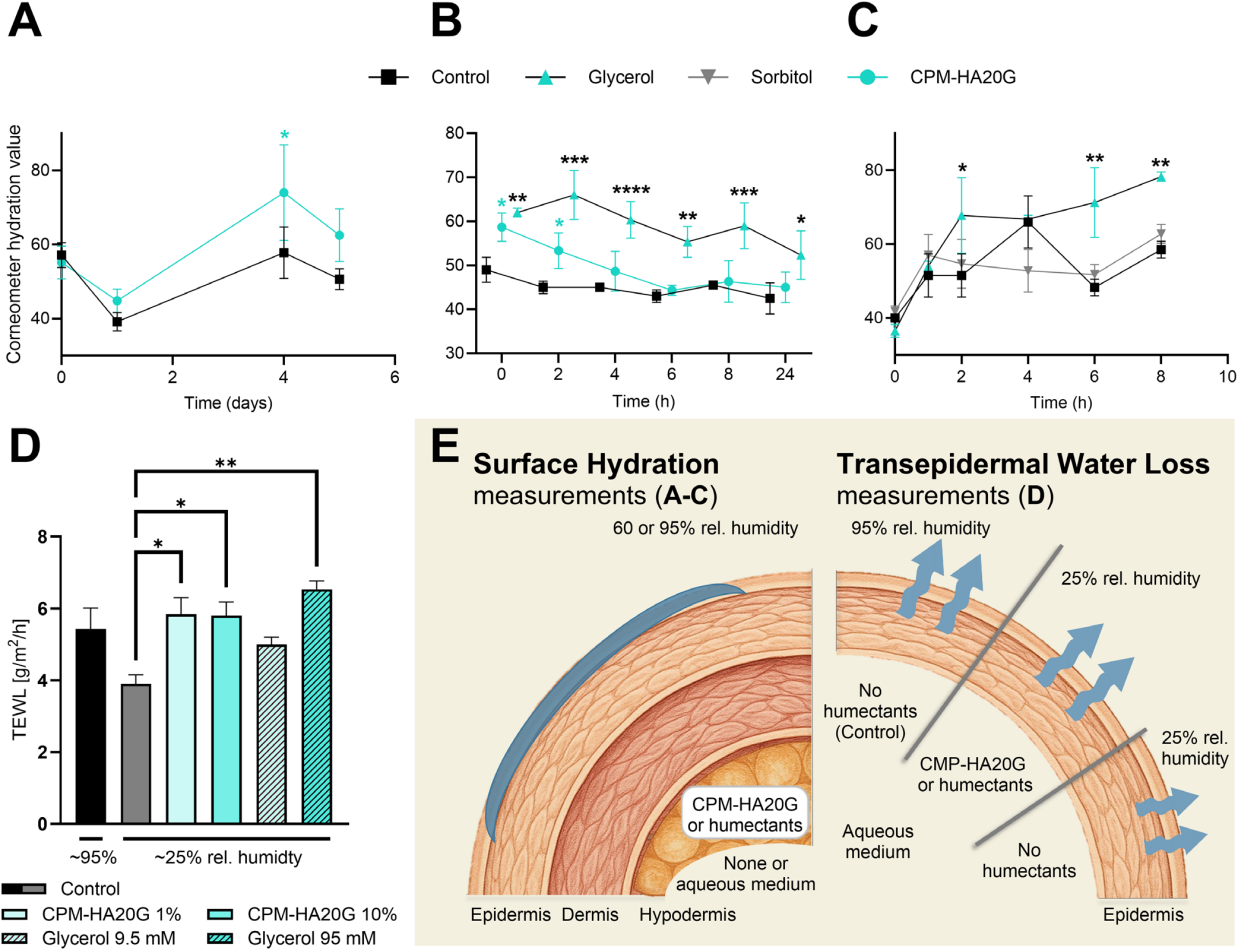
Superficial skin hydration and transepidermal water loss after treatment with CPM‐HA20G or glycerol. (A) Assessment of superficial skin hydration 100 μL bolus injection of CPM‐HA20G into a hydrated ex vivo human skin model (*n* = 3). (B) Comparison of superficial hydration effects following a 150 μL intradermal bolus injection of CPM‐HA20G or 5.4 M glycerol in a dehydrated ex vivo human skin model. (C) Comparison of superficial hydration after a 100 μL intradermal bolus injection of 5.4 M glycerol or 5.4 M sorbitol in a hydrated ex vivo human skin model. Control: PBS (*n* = 2). (D) Transepidermal water loss (TEWL) after systemic treatment with CPM‐HA20G (1%, 10%) or glycerol (9.5 mM, 95 mM) in a reconstructed human epidermis (RHE) model under dry conditions (25% rel. humidity). Control: Untreated RHE at 95% or 25% rel. humidity. Statistical analysis was performed using one‐way ANOVA with Dunnett's multiple comparison (A, D), two‐way ANOVA with Dunnett's multiple comparison (B, C). Significance denoted as: *p < 0.05; **p < 0.01; ***p < 0.001; ****p < 0.0001. (E) Schematic illustration of the experimental setup for surface hydration measured by corneometry in ex vivo human skin models (A‐C) and TEWL in RHE models (D).

Following inner hydration, the potential dynamics of surface hydration and water loss were investigated using transepidermal water loss analysis and corneometry on ex vivo skin models for extended time frames, as illustrated in Figure 2E. The surface hydration effect of CPM‐HA20G was examined comparing an intradermal bolus injection to a PBS control over a five‐day period. After 4 days, corneometry showed a peak in skin hydration with a significant 28% increase in hydration compared to PBS (*p* < 0.05) (Figure 2A). A dehydrated ex vivo skin model was further used to evaluate the hydrating effect of CPM‐HA20G compared to glycerol. Skin samples were injected intradermally with either CPM‐HA20G, 5.4 M glycerol, or PBS as a control. Corneometer hydration analysis showed that both glycerol and CPM‐HA20G significantly increased hydration compared to the average control by an average of 31% ± 11% (*p* < 0.001) and 10% ± 11% (*p* < 0.05), respectively (Figure 2B). This effect was maintained over 24 h.

To evaluate the specific hydrating effect of glycerol, it was compared to sorbitol, a widely used humectant in other dermal fillers. Two hours after injection, a higher hydration performance was observed for glycerol in comparison to sorbitol and control, and the significantly higher hydration values were constantly maintained in both cases over the remaining time (*p* < 0.01) (Figure 2C).

While the μCT analysis demonstrated that subcutaneous injection of CPM‐HA20G induced rapid hydration, the corneometry data additionally show that CPM‐HA20G provides a sustained increase in skin surface hydration over several days. Both CPM‐HA20G and glycerol significantly enhance skin hydration, with glycerol revealing a greater effect. Furthermore, the data indicate that glycerol may promote a more sustained hydrating effect compared to sorbitol, with a fast onset after 2 h.

Following the analysis of skin surface water content, transepidermal water loss (TEWL) was assessed, as the epidermal barrier plays a critical role in regulating water diffusion across the skin. TEWL measurements were conducted under conditions of standard (~95%) and low (~25%) relative humidity to evaluate changes in TEWL after treatment with CPM‐HA20G or glycerol.

Exposure to reduced relative humidity (~25%) resulted in formulation‐dependent modulation of TEWL. Compared to the low‐humidity control, treatment with CPM‐HA20G at concentrations of 1% and 10% significantly increased TEWL (*p* < 0.05), reaching levels comparable to those observed in untreated samples under standard relative humidity (~95%). This finding suggests a protective effect of TEWL values by CPM‐HA20G against dry conditions.

Similarly, glycerol exhibited a concentration‐dependent effect. Treatment with 9.5 mM glycerol resulted in moderately elevated TEWL, whereas 95 mM glycerol led to a significant increase in TEWL compared to the low‐humidity control (*p* < 0.01).

Overall, these results demonstrate that TEWL is influenced by ambient humidity and formulation concentration, with CPM‐HA20G and glycerol mitigating the effects of low humidity on epidermal water loss.

### 3.3 Glycerol Uptake in Keratinocytes Occurs Independent of AQP3 Modulation

**FIGURE 3:**
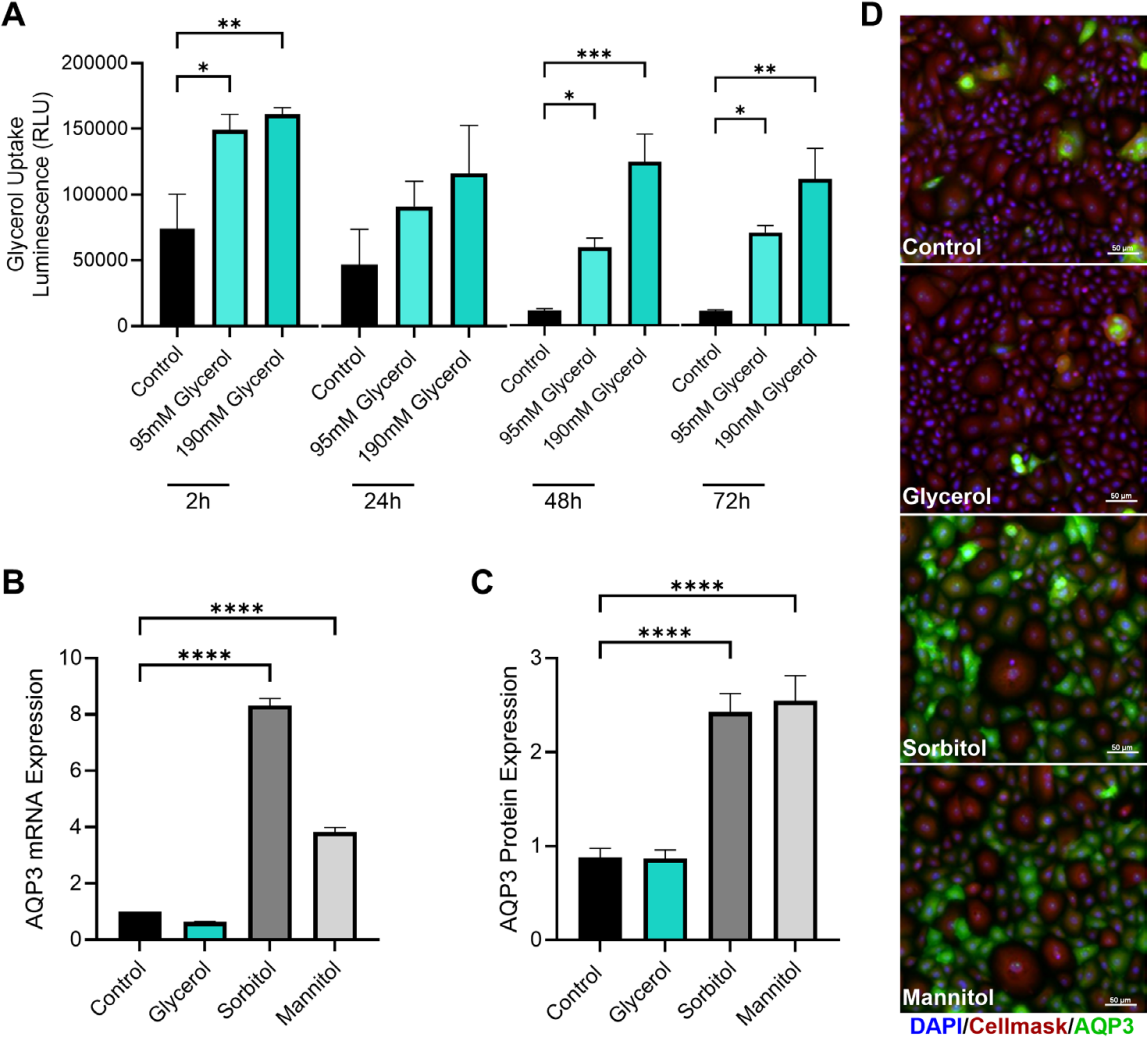
Glycerol uptake in keratinocytes. (A) Glycerol uptake into normal human keratinocytes in vitro after treatment with 95 or 190 mM of glycerol for 2, 24, 48, or 72 h using a bioluminescent assay. (B) Effect of glycerol, sorbitol, and mannitol on *AQP3* gene expression. (C, D) Visualization of AQP3 expression via immunocytochemistry and Quantification of AQP3 expression as measured by fluorescence intensity. (B, C) Treatment of cells was performed with 190 mM glycerol, sorbitol or mannitol. One‐way ANOVA with Tukey's multiple comparison, significance denoted as: *p < 0.05; **p < 0.01; ***p < 0.001; ****p < 0.0001. Nuclei were stained with DAPI (blue), cytoplasm with Cellmask (red), and AQP3 (green). Scale = 50 μm. Experiments were performed in biological triplicates (*n* = 3).

To understand the cellular interaction of glycerol with the outermost viable layer of the skin, the glycerol uptake by keratinocytes was assessed in vitro using a bioluminescence‐based quantification assay. Keratinocytes exhibited concentration‐dependent glycerol uptake, with a significant increase occurring after 2 h at both 95 and 190 mM (*p* < 0.05 and *p* < 0.01, respectively). Significant increases in glycerol uptake were also observed after 48 h (*p* < 0.05 and *p* < 0.001) and 72 h (*p* < 0.05 and *p* < 0.01) of treatment at both concentrations (Figure 3A). As AQP3 was reported to specifically mediate the transporter of glycerol and water into keratinocytes, AQP3 expression was analyzed. *AQP3* gene expression remained unchanged by glycerol uptake, as analyzed by qPCR (Figure 3B). In contrast, a significant increase in *AQP3* expression was observed with both sorbitol and mannitol treatments (*p* < 0.001). These findings were confirmed at the protein level via immunocytochemistry, with significantly increased AQP3 signal observed in keratinocytes treated with 190 mM sorbitol or mannitol (*p* < 0.001), whereas those treated with 190 mM glycerol showed similar levels of AQP3 expression as the control (Figure 3C,D).

Keratinocytes showed significant, concentration‐dependent uptake of glycerol without an increase in AQP3 expression, whereas sorbitol and mannitol treatments significantly upregulated AQP3 at the gene and protein levels.

### 3.4 Glycerol Is Well‐Tolerated in Keratinocytes

**FIGURE 4:**
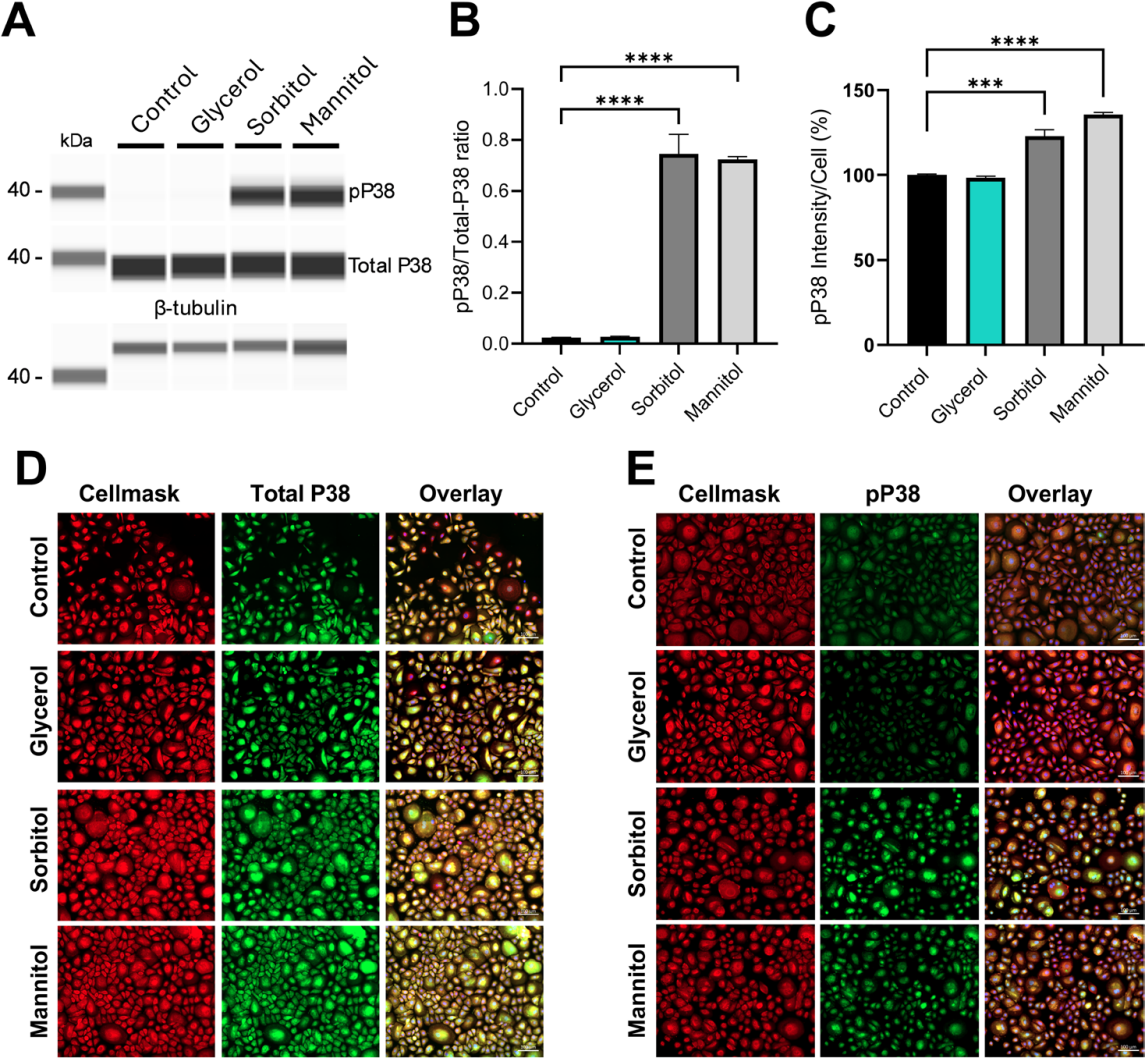
Cell tolerance to different humectants in keratinocytes. (A) Western blot showing protein expression of phosphorylated P38 (pP38) and total P38 after treatment with glycerol, sorbitol, or mannitol. Loading control: Beta‐tubulin. (B) Quantification of the phosphorylated pP38/total P38 ratio (indicating level of P38 activation), based on western blot signal intensity. (C) Quantification of pP38 protein expression based on immunofluorescence images showing total pP38 and P38 expression in keratinocytes after treatment with glycerol, sorbitol, and mannitol (D, E). Treatment of cells was performed with 190 mM glycerol, sorbitol, or mannitol. One‐way ANOVA with Tukey's multiple comparison, significance denoted as: ***p < 0.001; ****p < 0.0001. Scale = 100 μm. Experiments were performed in biological triplicates (*n* = 3).

To assess the cellular tolerability of humectants in keratinocytes, cells were treated with either glycerol, sorbitol, or mannitol at a concentration of 190 mM and analyzed for changes in cellular stress levels via pP38/tP38. While sorbitol and mannitol significantly increased phosphorylated‐P38 levels, indicative of cellular stress, glycerol treatment did not lead to any notable change as indicated in Figure 4A,B for western blot analyses and Figure 4C–E for immunofluorescence‐based quantification.

**FIGURE 5:**
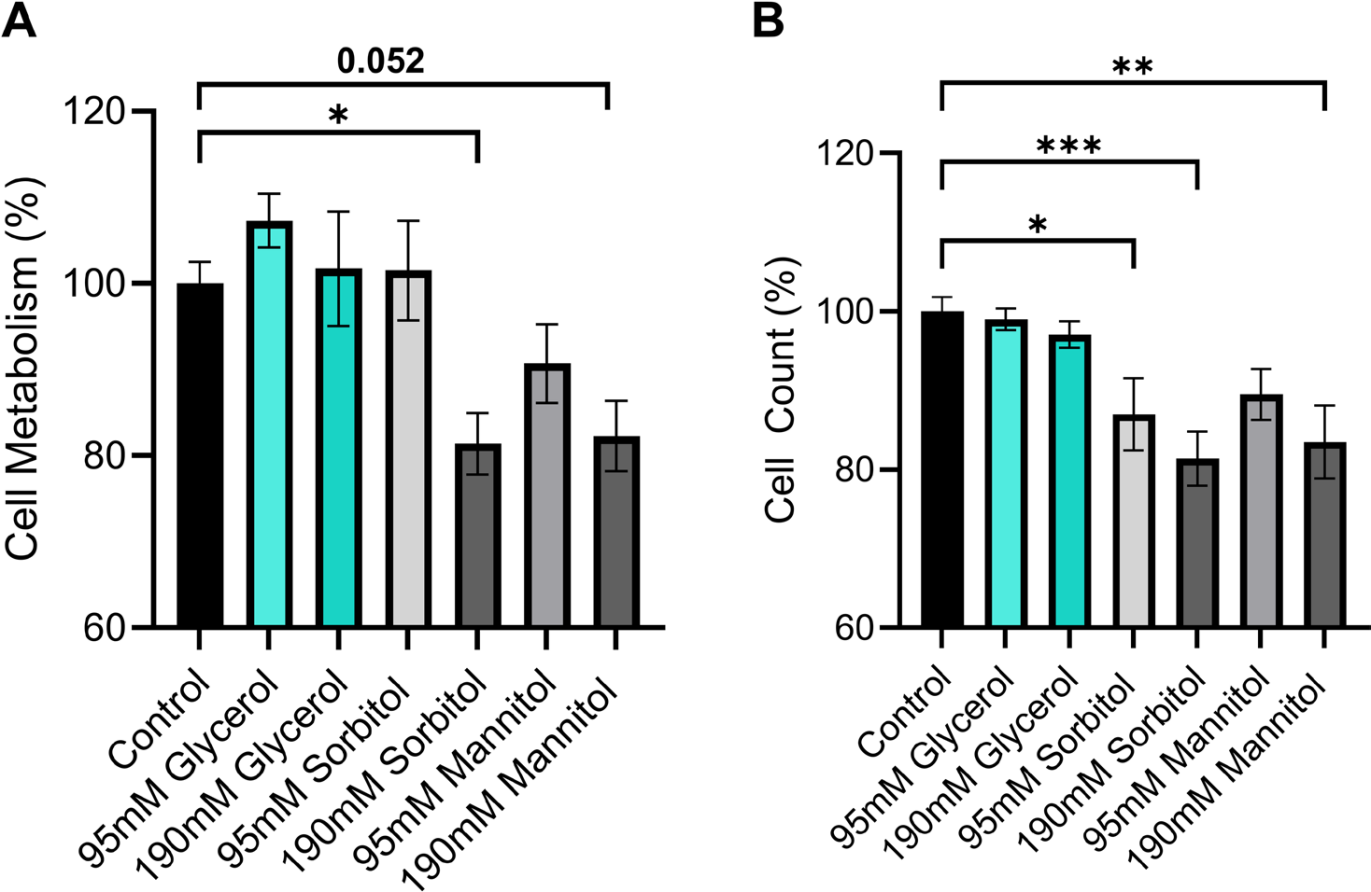
Comparative analysis of cell viability using different humectants. (A) Relative cell metabolism in keratinocytes after 24 h. Data were normalized to control (untreated); (B) Cell counts after treatment with 95 or 190 mM of glycerol, sorbitol, or mannitol. Data were normalized to control (untreated). One‐way ANOVA with Tukey's multiple comparison, significance denoted as: *p < 0.05; **p < 0.01; ***p < 0.001; ****p < 0.0001. Experiments were performed in biological triplicates (*n* = 3).

Glycerol treatment at 95 and 190 mM also had no significant effect on cell metabolism, whereas the 190 mM sorbitol treatment showed a significant reduction (*p* < 0.05) (Figure 5A). Mannitol treatments also reduced cell metabolism; however, this effect was not statistically significant. Similarly, both sorbitol treatment concentrations and the 190 mM mannitol treatment significantly reduced cell number (*p* < 0.05, *p* < 0.001, and *p* < 0.01, respectively), whereas glycerol showed no significant change (Figure 5B).

Together with the assessment of phosphorylated p38 levels, these data reveal that, unlike sorbitol and mannitol, glycerol did not induce cellular stress and does not impair cell viability.

Inhibition of glycerol transport via aquaporins significantly modulated p38 MAPK signaling. Treatment with DFP (50–100 μM) increased the pP38/tP38 ratio compared to control indicating activation of stress pathways. This effect was partially reversed by glycerol (95–190 mM) (*p* < 0.01, *p* < 0.001), suggesting compensatory effects for reduced intracellular glycerol (Figure S1A).

Similarly, phloretin (250 μM) elevated the pP38/tP38 ratio (*p* < 0.001), and co‐treatment with glycerol reduced this activation in a concentration‐dependent manner (*p* < 0.05) (Figure S1B). Overall, AQP inhibition appears to limit glycerol uptake, triggering p38 MAPK activation, while extracellular glycerol partially restores cellular balance.

### 3.5 Glycerol Protects and Rescues Against Exogenous Stress

**FIGURE 6:**
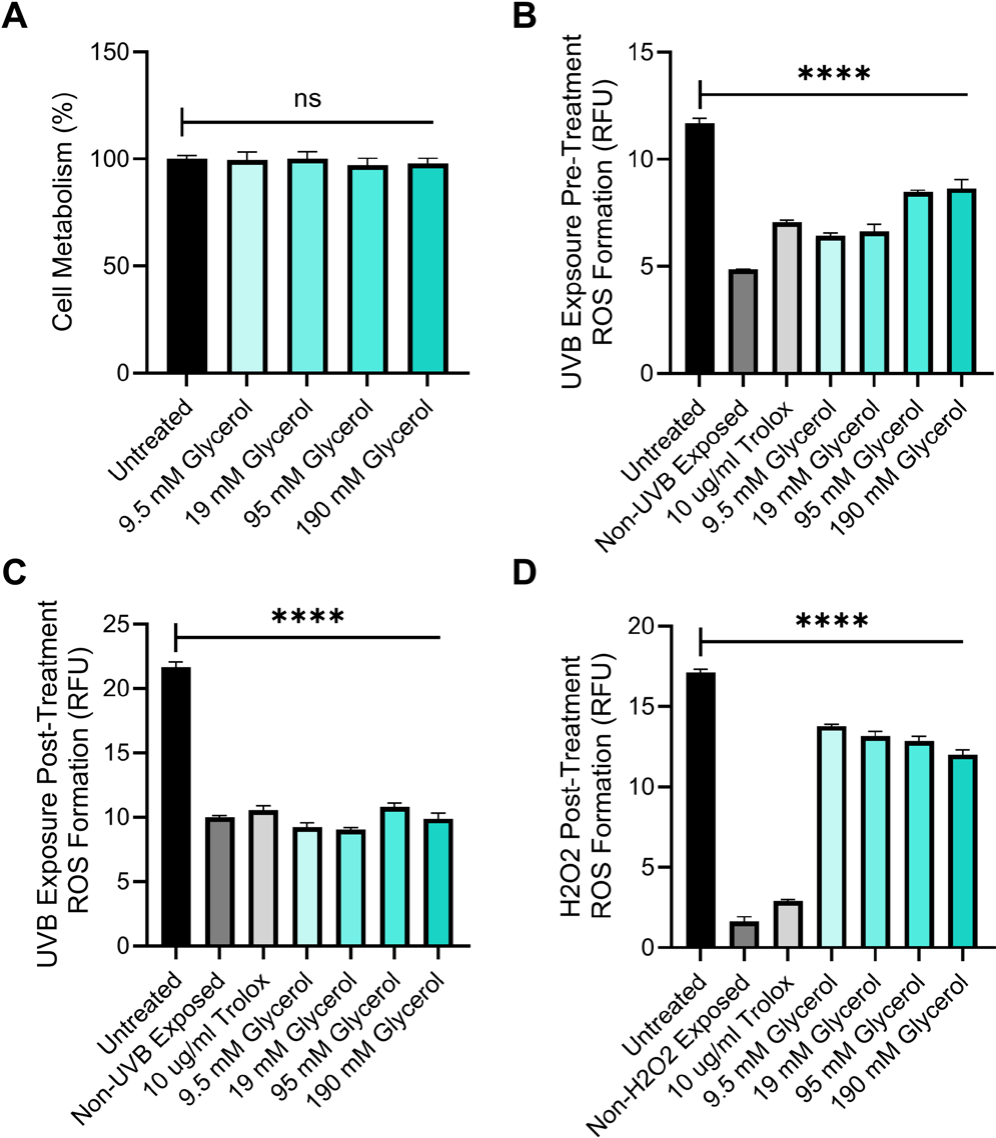
ROS formation in keratinocytes after exposure to exogenous stress. (A) Initial assessment of cell metabolism with different glycerol concentrations for ROS formation assay after 24 h. (B) ROS formation after pre‐treatment with 190, 95, 19, 9.5, and 1.9 mM glycerol followed by exposure to 100 mJ/cm^2^ UVB. (C) ROS formation after UVB exposure and subsequent treatment with a series of glycerol concentrations. (D) ROS formation after exposure to 200 μM H_2_O_2_ and subsequent treatment with glycerol. Positive control: 10 μg/mL Trolox. One‐way ANOVA with Tukey's multiple comparison, significance denoted as: Ns = not significant; *p < 0.05; **p < 0.01; ***p < 0.001; ****p < 0.0001. Experiments were performed in biological triplicates (*n* = 3).

Following the analysis of cellular tolerability of glycerol, potential protective or rescuing effects by glycerol against ROS in keratinocytes were analyzed. Keratinocytes were either pre‐treated with glycerol before UVB exposure or treated with glycerol after UVB exposure. Trolox, an analogue of vitamin E, was used as a positive control for a protective compound against ROS formation. UVB exposure significantly increased ROS formation in keratinocytes compared to non‐UVB‐treated controls (*p* < 0.0001). Pre‐treatment with all concentrations of glycerol significantly protected against ROS formation compared to UVB‐exposed controls, with lower ROS levels observed as glycerol concentration increased (Figure 6B). Similarly, treatment with all concentrations of glycerol after UVB exposure significantly reduced ROS formation compared to UVB‐exposed controls (*p* < 0.0001) (Figure 6C).

Cells were also treated with the same concentrations of glycerol before being exposed to 200 μM H_2_O_2_. Here, glycerol exhibited protective effects against ROS, resulting in a significant reduction in ROS formation compared to H_2_O_2_‐exposed controls (*p* < 0.0001) (Figure 6D).

The data indicate that glycerol provides both protective and rescuing effects against UVB, when administered before or after exposure. Similarly, glycerol provides a rescuing effect against H_2_O_2_ mediated ROS formation.

### 3.6 CPM‐HA20G Promotes Tissue Integration While Minimizing Inflammatory Response

In the *vivo* study, the HA and glycerol dermal filler CPM‐HA20G was assessed in two key performance indicators: tissue integration and inflammatory response. CPM‐HA20G and HA12.5S, a cross‐linked HA filler which contains the humectant sorbitol, were injected via intradermal bolus injection. The assessments were conducted two weeks post‐injection.

**FIGURE 7:**
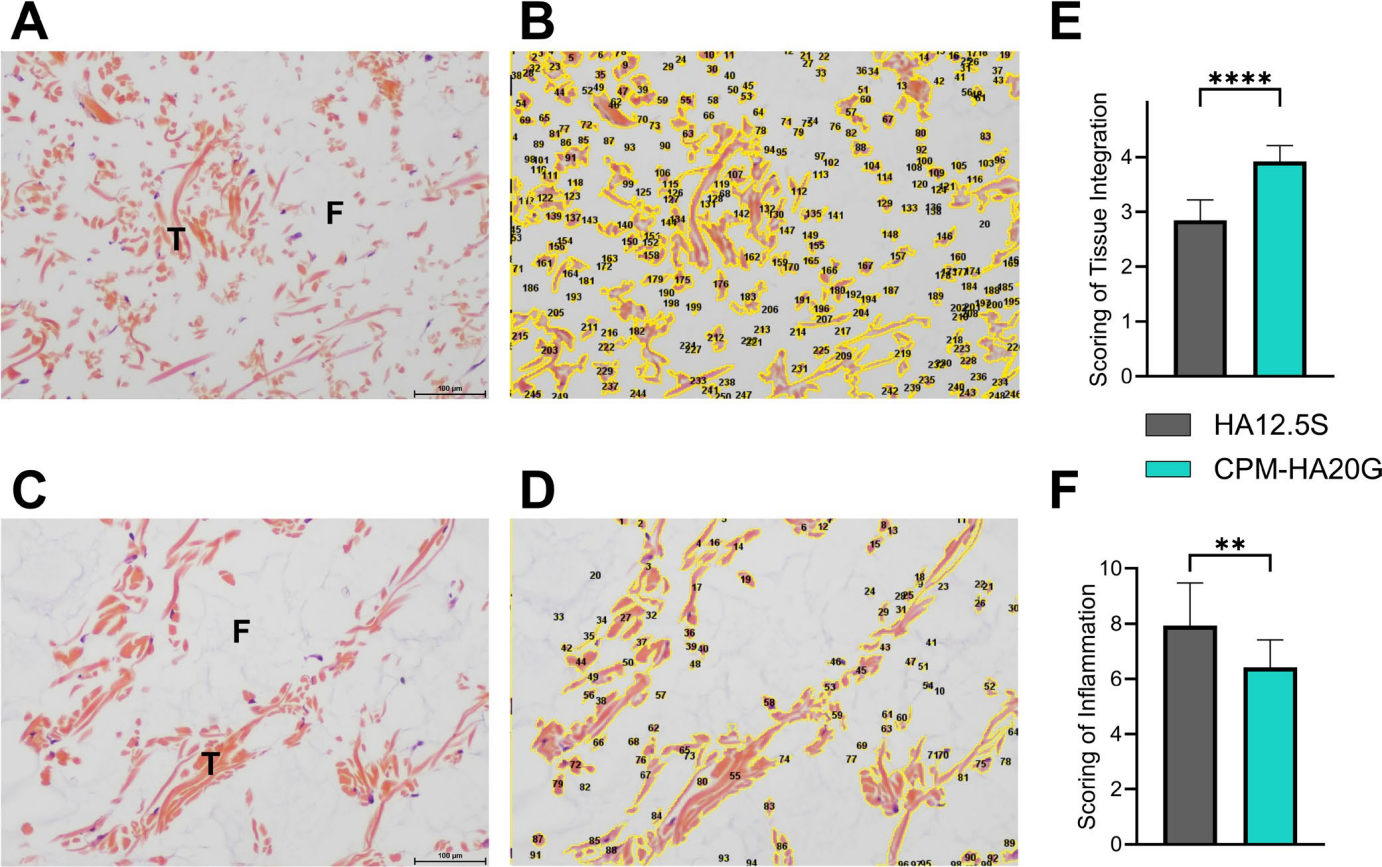
Tissue integration and inflammation after HA filler injection. (A) SHE staining of histological sections (~4.5 μm) immediately after CPM‐HA20G (*n* = 12) injection or (C) HA12.5S (*n* = 13) injection. Intradermal bolus injections (200 ± 40 μL). (B, D) Respective tissue segment quantification. (E) Semiquantitative analysis of tissue integration and (F) inflammation scoring by DESV‐Pathology after two weeks. Unpaired t‐test, significance denoted as: **p < 0.01; ****p < 0.0001. F = filler; T = tissue; Scale = 100 μm.

Tissue integration of a filler can be defined as the process by which the injected material becomes physically incorporated homogenously into the dermis, entangling within the surrounding collagen and elastin fibers [13]. CPM‐HA20G displayed a homogenous distribution within the tissue, with few large accumulations of product between collagen bundles in comparison to HA12.5S (Figure 7A vs. 7C). This was reflected in a greater number of tissue segments for CPM‐HA20G (245 ± 6 for CPM‐HA20G vs. 123 ± 27 for HA12.5S) (Figure 7B,D).

Histological H&E analysis demonstrated that CPM‐HA20G exhibited nearly complete tissue integration. with a significantly higher semi‐quantitative integration score of 3.9 ± 0.3 compared to a score of 2.8 ± 0.4 for HA12.5S (*p* < 0.0001) (Figure 7E).

Sites injected with CPM‐HA20G or HA12.5S demonstrated low immune cell infiltration (Figure 7F). The inflammation response was characterized predominantly by a “slight reaction” in terms of macrophages and lymphocytes and “slight to moderate reaction” in terms of polymorphonuclear cell numbers. Consequently, the overall reactivity inflammation score for both fillers was classified as slight. However, CPM‐HA20G exhibited a significantly lower inflammatory score (6.4 ± 1.0) compared to HA12.5S, a sorbitol‐containing HA filler (7.9 ± 1.5; *p* < 0.01).

## 4 Discussion

This study offers mechanistic insights into the dual hydration capabilities of CPM‐HA20G, revealing novel pathways through which glycerol‐enhanced hyaluronic acid formulations promote skin quality parameters. The findings establish a scientific foundation for understanding the clinical outcomes observed in previous studies and position glycerol as an important bioactive component in next‐generation dermal fillers.

**FIGURE 8:**
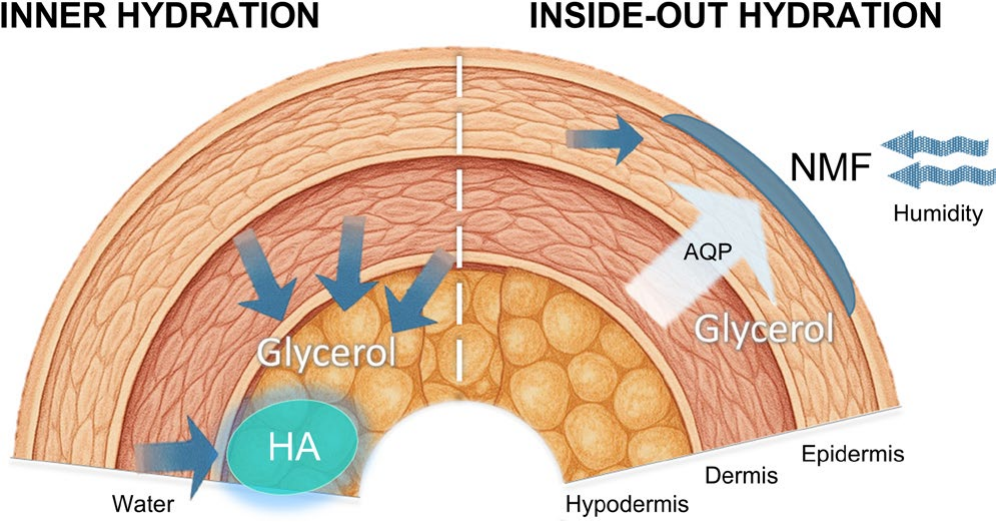
Schematic presentation of the dual hydration mechanism of CPM‐HA20G in the skin. The diagram illustrates two complementary hydration pathways acting across the epidermis, dermis, and hypodermis. The inner hydration mechanism (left) shows the penetration of CPM‐HA20G–derived glycerol and HA into deeper skin layers, where hyaluronic acid (HA) retains water within the extracellular matrix and glycerol acting as a natural humectant, enhancing internal moisture storage. The inside‐out hydration mechanism (right) depicts the transport of glycerol and water through aquaporin (AQP) channels toward the upper epidermis, promoting water diffusion from deeper layers to the stratum corneum. Concurrently, natural moisturizing factors (NMF) attract and bind atmospheric humidity at the skin surface, reinforcing moisture retention. Together, these mechanisms provide both deep reservoir hydration and sustained surface moisturization. AQP: Aquaporin, HA: Hyaluronic acid, NMF: Natural moisturizing factors.

The present study elucidated two complementary hydration mechanisms that contribute to the clinical efficacy of CPM‐HA20G: the immediate “inner” hydration and progressive “inside‐out” hydration (Figure 8). The initial inner volumetric expansion of hydrated tissue, as measured by μCT, is driven by the synergistic action of HA and glycerol. Within 2 h, the volume of the affected tissue exceeded the initially injected volume, demonstrating rapid water influx and tissue expansion.

In addition, μCT analysis revealed that volume expansion at the injection site was associated with increased HU values. This increase likely reflects the influx of culture medium and enhanced tissue hydration, resulting in higher radiodensity compared with the hypodermis. Variability in baseline HU values at early time points is attributable to injection‐related factors, including differences in injected volume and local tissue heterogeneity. Factors such as injection depth and interactions with surrounding structures may further influence local X‐ray attenuation and HU baseline shifts.

Analysis of the surface hydration also showed that ex vivo skin models treated with glycerol exhibited significantly higher hydration levels compared to those treated with sorbitol or PBS, indicating a functional role of glycerol as a humectant. This is in line with the literature of Fluhr et al., summarizing glycerol as being among the most effective humectant polyols [14].

While cross‐linked HA remains localized, glycerol can freely diffuse through the subcutaneous, dermal, and superficial layers, acting as a mediator of water influx to the injection site, enhancing overall tissue hydration. The subsequent “inside‐out” hydration effect is mediated through the concentration‐dependent uptake of glycerol in keratinocytes. Glycerol contributes to hydration by diffusing into the dermis and penetrating the superficial layers of the epidermis via functional aquaporin channels of keratinocytes, which are known to be essential for its transit activity [15]. In the superficial layers of the skin, glycerol acts as one of the Natural Moisturizing Factors (NMFs), contributing to hydration maintenance [16]. This function is largely attributed to its hygroscopic nature and molecular structure, which enable it to form strong hydrogen bonds with water molecules [17]. Under dehydrated conditions, glycerol's capacity to retain water within the epidermis and attract environmental humidity further increased its hydrating potential [18]. The observed increase in superficial hydration, particularly in dry skin models, is attributable to this mechanism. The inside‐out mechanism supports the concept that superficial hydration induced by glycerol is independent of perforation caused by needle injection in clinical settings. Together, the two complementary hydration mechanisms offer a comprehensive explanation for the rapid improvements in skin hydration and glow observed in clinical treatments [19].

A key regulator of epidermal glycerol dynamics is AQP3, a membrane channel highly expressed in keratinocytes. AQP3 facilitates the transport of water and glycerol across cell membranes and into the upper layers of the skin [15, 20]. Notably, glycerol was taken up by keratinocytes without altering AQP3 expression. Functional studies in AQP3‐deficient mice have demonstrated that loss of this transporter leads to a marked reduction in SC hydration, skin elasticity, and delayed barrier repair, effects that correlate with decreased epidermal glycerol levels rather than alterations in SC structure or lipid content [21]. Notably, these deficits are specifically reversed by topical or systemic glycerol administration, whereas other osmolytes such as xylitol, erythritol, or urea do not exert the same effects [21, 22].

Dysregulation of aquaporins has been implicated with pathology of skin diseases, inflammation and impaired skin barrier function indicating that artificial interventions into the expression profile of aquaporins are undesirable [23]. Glycerol as natural humectant did not change AQP3 expression levels in contrast to Sorbitol and Mannitol, both increasing AQP3 mRNA levels, thereby inducing an unwanted dysregulation and artificial influence on AQP3 channels. Beyond its role in hydration, glycerol has been implicated in regulating keratinocyte function, with mechanistic investigations demonstrating that glycerol serves as a substrate supply for phospholipase D2 (PLD2), leading to the generation of phosphatidylglycerol [20]. This bioactive lipid promotes early keratinocyte differentiation and contributes to epidermal homeostasis [20, 24]. The current work demonstrates the antioxidant properties of glycerol following treatment with exogenous noxae, indicating the protective effects of CPM‐HA20G against for example, photodamage. In particular, the ROS reduction experiments with UVB‐exposure provide a molecular basis for the product's indication in the treatment of early‐onset photodamage. This antioxidant mechanism, combined with glycerol's role in supporting keratinocyte differentiation through PLD2 activation and phosphatidylglycerol generation, suggest beneficial roles associated with anti‐aging intervention that addresses multiple pathways of skin deterioration [25]. Moreover, while the alternative humectants, sorbitol and mannitol activated P38 stress signaling and compromised cell viability, glycerol preserved cellular health, demonstrating both tolerability and physiological compatibility.

Recently, CPM‐HA20G was shown to reduce TEWL values in subjects with healthy skin at weeks 12 and 24, indicating an improvement in skin barrier function [19]. Here, within a dry skin model, TEWL values remained consistent with the TEWL score of the control under standard humid conditions, suggesting a protective effect of CPM‐HA20G and glycerol on epidermal barrier dynamics. This observation may be explained by the presence of glycerol, which has been reported to enhance lipid cohesion within the stratum corneum, thereby supporting barrier stability and normalization of TEWL under dry conditions [5]. It would therefore be of interest to evaluate whether a comparable regulatory effect on TEWL and skin barrier function can be demonstrated in a clinical population of subjects with dry skin.

The observed tissue integration, coupled with a comparatively low inflammatory response, suggests that the biocompatibility of CPM‐HA20G extends beyond hydration properties alone to also influence tissue remodeling processes. CPM‐HA20G and HA12.5S differ in their humectant composition, with humectants that may interact differently within the crosslinked HA and influence local tissue interactions [26]. In addition, the formulations differ in physicochemical characteristics, for example, HA content or crosslinking technologies, which may contribute to different gel behavior and tissue response. These observations are consistent with recent meta‐analyses and systematic reviews, demonstrating that HA‐based fillers are associated with improvements in skin hydration and radiance while providing mechanistic explanations for the differential performance observed among various formulations [27, 28]. The observed tissue integration further corresponds with clinical reports of natural‐appearing outcomes and favorable patient satisfaction. The homogenous distribution pattern and reduced tissue condensation suggest that CPM‐HA20G may support controlled gel placement, which is consistent with the safety profile reported in clinical applications [7, 29].

This study has several limitations that should be considered when interpreting the findings. The findings are primarily based on ex vivo human skin models and short‐term observations, which do not fully reflect the complexity of living skin. Consequently, systemic effects and long‐term responses cannot be assessed, limiting direct translation to in vivo clinical efficacy. All skin samples were derived from Caucasian donors with Fitzpatrick skin type III, which may limit the generalizability of the findings to other skin types with different structural and hydration characteristics. The short observation period restricts insights into the durability and long‐term dynamics of the observed effects. Hydration‐related changes, barrier recovery, and metabolic responses may evolve over extended timeframes that were not captured in the present study.

Finally, the study compared only a limited number of humectants, which may not fully reflect the diversity of moisturizing agents. The conclusions regarding relative performance and mechanisms should be interpreted within the context of the specific compounds tested.

Building on the insights generated from this work, future research will focus on translating the promising ex vivo and mechanistic findings into clinically relevant outcomes. Key objectives include validating early‐stage improvements in skin hydration using in vivo measurement techniques that align with ex vivo readouts, thereby strengthening translational relevance. In addition, metabolomic profiling and tape‐stripping methodologies will be employed to gain deeper insight into hydration‐related mechanisms at the skin surface and within the stratum corneum [25, 29]. Furthermore, future clinical studies will investigate glycerol's antioxidative properties, building on its demonstrated protective effects against oxidative stress in experimental models. Together, these efforts aim to bridge the gap between mechanistic understanding and real‐world clinical applicability.

CPM‐HA20G offers a multifaceted approach to improving skin quality. In addition to cross‐linked HA, it uses the naturally occurring humectant glycerol to provide hydration through a dual hydration mechanism that acts both within and on the skin surface. The endogenous AQP‐permeable nature of glycerol allows it to hydrate effectively and protect skin cells from oxidative stress. Compared to other humectants, glycerol is well tolerated by cells. CPM‐HA20G also demonstrates good tissue integration, supporting its role as a tissue compatible and effective option for enhancing skin hydration, antioxidative capacity, and overall skin quality.

## Author Contributions

K.M., C.H., S.H.‐J. designed and conceived the study. K.M., C.H., D.S., C.W. performed experiments. S.H.‐J., K.M., C.H. analyzed the data. K.M., C.H., S.S., J.‐Y.P., T.S.L., L.A.P.H, S.H.‐J, T.H. wrote and reviewed the manuscript. All authors reviewed the results and approved the final version of the manuscript.

## Funding

This study was supported by Merz Aesthetics.

## Ethics Statement

Ex vivo human skin was used with the informed consent from donors and in compliance with the Declaration of Helsinki and all relevant institutional ethical guidelines. No patient‐identifiable data were included. In vivo study was conducted in an accredited AAALAC facility with authorization from the ethical committee (APAFIS #23401‐2 017 050 517 312 149 v12).

## Conflicts of Interest

The authors declare no conflicts of interest.

## Supporting information


**Table S1:** Similar osmolarity profile of the tested humectants.

**Figure S1:** Inhibition of AQP‐mediated glycerol transport by DFP and phloretin induces p38 MAPK activation, which is partially rescued by glycerol supplementation.

**Video S1:** μCT analysis at the injection site after CPM‐HA20G injection. 3D computer tomography analysis after subcutaneous bolus injection of CPM‐HA20G at 120 min post‐injection in a human skin model. Injection site: Green; Hypodermis: Gray; Epidermis/Dermis: Red.

## Data Availability

The data that support the findings of this study are available from the corresponding author upon reasonable request.
