# Supplementary material for: Reviving the Skin From Within: Mechanistic Insights Into a Well‐Tolerated Dermal Filler—CPM‐HA20G
Source: J Cosmet Dermatol. 2026 Jun 18;25(6):e70996. doi: 10.1111/jocd.70996 (PMC13280165; doi:10.1111/jocd.70996)
Supplement: Supplementary file 1 — Table S1: Similar osmolarity profile of the tested humectants. [file JOCD-25-e70996-s001.docx]

**Supplementary material:**

**Table S1:** Osmolarity of different materials at different concentrations.

| **Substance** | **Concentration (mM)** | **Mean ± SD Osmolarity (mOsm/L)** |
| --- | --- | --- |
| Glycerol | 19 | 354 ± 1 |
|  | 95 | 473 ± 35 |
|  | 190 | 572 ± 42 |
| Sorbitol | 19 | 355 ± 2 |
|  | 95 | 438 ± 3 |
|  | 190 | 537 ± 1 |
| Mannitol | 19 | 354 ± 2 |
|  | 95 | 433 ± 1 |
|  | 190 | 540 ± 1 |
| Cell Culture medium | - | 353 |
