# Supplementary material for: Reviving the Skin From Within: Mechanistic Insights Into a Well‐Tolerated Dermal Filler—CPM‐HA20G
Source: J Cosmet Dermatol. 2026 Jun 18;25(6):e70996. doi: 10.1111/jocd.70996 (PMC13280165; doi:10.1111/jocd.70996)
Supplement: Supplementary file 2 — Figure S1: Inhibition of AQP‐mediated glycerol transport by DFP and phloretin induces p38 MAPK activation, which is partially rescued by glycerol supplementation. [file JOCD-25-e70996-s002.docx]

**Supplementary material:**


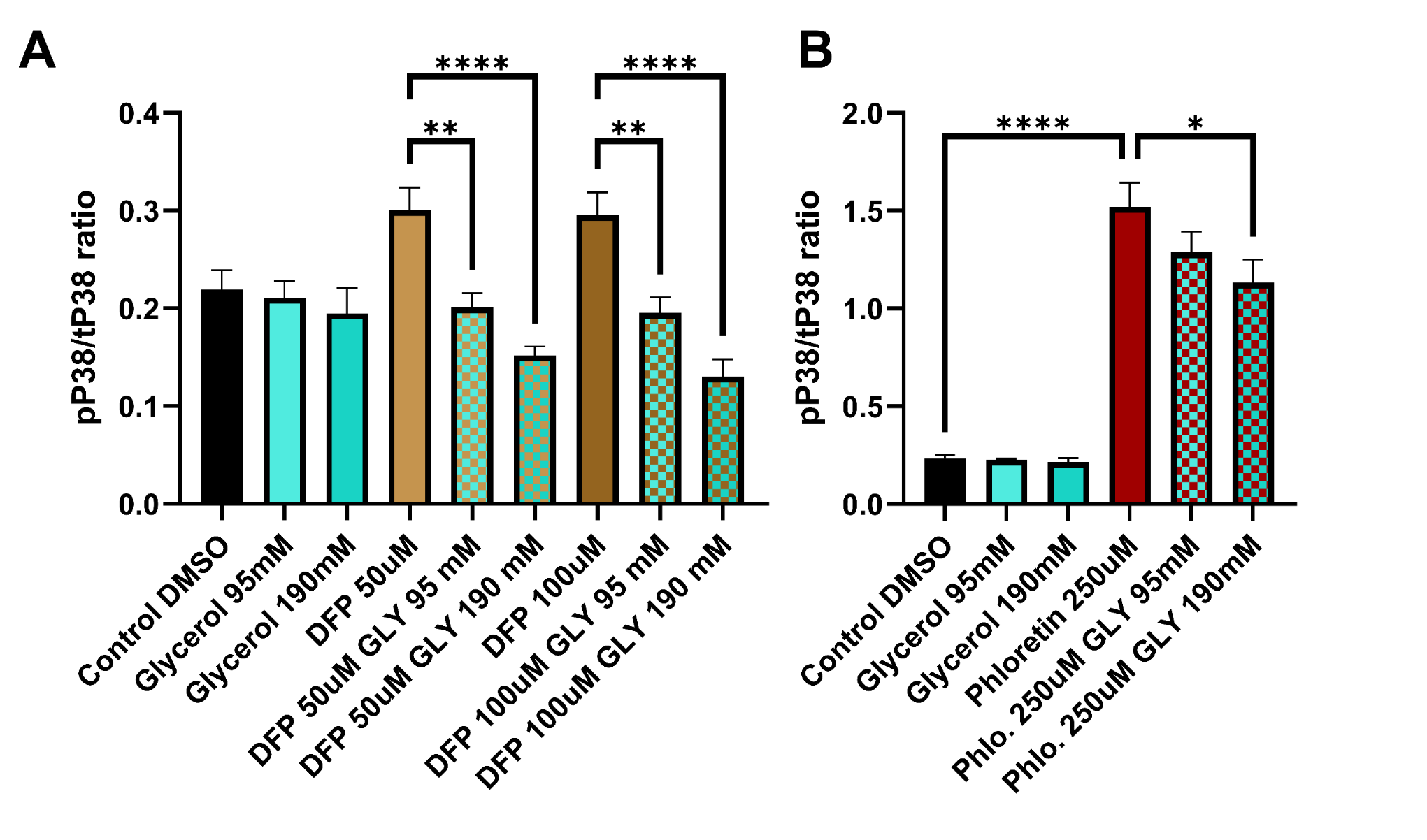


**Figure S1: Inhibition of AQP-mediated glycerol transport by DFP and phloretin induces p38 MAPK activation, which is partially rescued by glycerol supplementation.** (A) pP38/tP38 ratios following treatment with DFP (50-100 µM), an inhibitor of AQP3-dependent glycerol transport, in the presence or absence of glycerol (95–190 mM). (B) pP38/tP38 ratios after treatment with phloretin (250 µM) impaired AQP-mediated glycerol uptake. Experiments were performed in biological triplicates (n=3). Data are presented as mean ± SEM. Statistical significance is indicated as *p < 0.05, **p < 0.01, ***p < 0.0001.
